# 

**Published:** 1881-01

**Authors:** 


### INDEX TO VOLUME TWENTY-EIGHT.

### A

| | Page. |
|---|---|
| Antidotes to Snake Poison | 451 |
| Accidents and Emergencies | 13 |
| Atmospheric Dust | 28 |
| Athletic Exercise | 47 |
| A Suggestion to Manager Abbey | 124 |
| A Cool Detective | 168 |
| A Physician's Life-time | 180 |
| Abolish the Use of Liquors | 243 |
| A New Fashion of Sleeping | 273 |
| A Happy Home | 304 |
| Anti-fat | 320 |
| A Ship's Log | 326 |
| Apoplexy | 333 |
| Auscultation | 365 |
| A Monster of the Sea | 368 |
| A Habit of Sleep | 373 |
| A Cure by Imagination | 374 |
| Aims of the Journal | 379 |
| Advantages of Sun-light | 392 |
| A Sign of Consumption | 480 |
| About Love | 100 |
| A Cure for Sea-sickness | 112 |
| A Great River in Alaska | 123 |
| A Reflection (poetry) | 128 |
| Annual Ailments | 135 |
| Appetite | 175 |
| A Cure for Car-sickness | 236 |
| Anæmia | 403 |
| Anti-Monopoly | 406 |
| A Cure for Small-pox | 432 |
| About Gondolas | 448 |

### B

| | |
|---|---|
| Burying Alive | 10 |
| Beautiful Snow (poetry) | 80 |
| Bad Temper and Insanity | 215 |
| Baths and Bathing | 287 |
| Blinders | 336 |
| Breakfast-Table Talk | 99 |
| Be Systematic | 179 |
| Benefit of Washing in Cold Water | 372 |

### C

| | Page. |
|---|---|
| Cancer | 9 |
| Congestion | 84 |
| Creosote for Bronchitis and Catarrh | 34 |
| Care of the Hair | 94 |
| Cancer | 111 |
| Clothing and Diet of Children | 49 |
| Causes of Disease | 119 |
| Cold Feet | 129 |
| Coughing | 140 |
| Climbing the Ladder of Fame | 170 |
| Climate for Consumption | 185 |
| Carelessness | 186 |
| Cold Feet | 226 |
| Characteristics of Doctors and others | 267 |
| Color of Lightning | 294 |
| Catarrh | 311 |
| Corns Cured | 382 |
| Contagious Diseases | 401 |
| Concerning Swimming | 411 |
| Consumption | 453 |
| Curious Facts | 449 |

### D

| | |
|---|---|
| Danger from Decaying Teeth | 440 |
| Dyspepsia | 3 |
| Does Eucalyptus prevent Fever ? | 67 |
| Dieting | 45 |
| Diseases of the Kidneys | 79 |
| Difference between Cousins | 103 |
| Decision of Character | 176 |
| Decency toward Horses | 209 |
| Dead in the Saddle | 210 |
| Dieting for Health | 214 |
| Dollars and Ideas | 224 |
| Dangerous Newspaper Receipts | 260 |
| Debt and Death | 265 |
| Don't Drown with a Shingle within reach | 391 |
| Don't take it to Heart (poetry) | 404 |

### E

| | |
|---|---|
| Educated Girls | 167 |
| Eating, Drinking and Exercise | 444 |
| Eyebrows | 371 |

### F

| | |
|---|---|
| Five Weeks in a Trance | 110 |
| From an Insect's Point of View | 207 |
| Fashionable Freaks | 313 |
| Facts for the Curious | 357 |
| Flower Pots | 426 |
| Facts about Gold | 450 |

### G

| | |
|---|---|
| God's Decree (poetry) | 203 |
| Glucose | 291 |
| Good Night (poetry) | 292 |
| Grow Beautiful | 378 |
| Going to the South | 382 |
| Garden and Farm | 452 |

### H

PAGE.
How to Get a Wife in India ... 61
Habit ... 134
House-Building ... 182
Health Ruined ... 189
How to Get Along ... 247
How to Hang Pictures ... 272
Health Maxims ... 397
How to Eat Lemons ... 316
How to Behave at a Party ... 318
Health and Disease ... 337
How to Keep Cool ... 387
How Sponges are Caught ... 430
How to Keep Your Friends ... 436
How to Sleep Well ... 445
Healthfulness of Fruit ... 473
How to Keep Warm ... 96
Heart Affections ... 131
H[illegible]l h ... 139
Hydrophobia ... 183
Horseback Riding ... 201

### I

Iron Smelting ... 39
Ice Water ... 141
In the West Indies ... 328
Is Alcohol Food ? ... 330
Importance of Mineral Elements of Food ... 408
Items ... 434
Interesting Facts ... 481
Influence of Christianity on Medical Science ... 484
Imaginary Diseases ... 81
In Sunset Light (poetry) ... 441

### L

Living in Italy ... 130

Lumber Supply of Washington Territory ... 59

Lead Colic ... 306

Lives of Medical Men ... 308

Latent Insanity ... 471

Lafayette at Yorktown ... 449

### M

Malaria (poetry) ... 46
Manifest Destiny of the English Race ... 62
Men and their Children ... 65
Muscular Exercise ... 82
My Old House (poetry) ... 98
Moderation in All Things ... 120
Mental Epidemics ... 257
Major Andre's Remains ... 275
Medicinal Qualities of Buttermilk ... 291
Moonlighting ... 293
Metamorphoses ... 310
Malaria ... 325
My Ship at Sea (poetry) ... 367
Milk ... 381

### N

New and Stale Bread ... 66

Neuralgia ... 163

Natural Death ... 253

Nature's Methods ... 263

Near Sighted ... 483

New Treatment of Diphtheria ... 483

Nursing the Sick ... 2[illegible]4

### O

PAGE.

Old Young People and Young Old People ... 7

Oxygen ... 17

Origin of Merino Sheep ... 51

Origin of the Morgue ... 63

One and Then Another (poetry) ... 295

Out-door Safety ... 133

Our Friends Beyond ... 252

Our Changing Climate ... 253

Origin of Diphtheria ... 271

### P

Praise Your Wife ... 18
Principles of Medical Practice ... 42
Poetry ... 85
Punctuality ... 127
Petroleum in Bronchitis ... 194
Poisons ... 264
Power of the Plug-hat ... 272
Position of the Body ... 285
Paper Plates ... 314
Physical Education of Children ... 385
Punishment by the Cangue in China ... 393
Popular Fallacies ... 425–474

### Q

Queer Industries in New York ... 128

### R

Remarkable Operations ... 444
Robert Burns (poetry) ... 59
Regimen ... 142
Reading ... 181
Removal of Stains and Spots ... 211
Riding on the Rails ... 233
Relations of Science to Speculation ... 355
Reserved Force ... 407
Reason in All Things ... 427

### S

Superstitions about Cats ... 60
Sprains ... 132
Small-pox ... 144
Suppressing the Mosquito ... 196
Short Talk on Protection ... 208
Straw Lumber ... 233
Signs of Disease ... 241
Spanish Murders and Brigandage ... 244
Seth Green's Spider Story ... 295
Some of the Great Bridges ... 313
Silk-worms at Work ... 319
Snuff-taking ... 352
Scientific Scraps ... 360
Scarlet Fever ... 375
Summer Complaint ... 388
Shaving the Beard ... 428
Sleeplessness and Insanity ... 446
Snow at Great Altitudes ... 107
Sleeping ... 143
Signs of Death ... 173
Sixteen and Sixty (poetry) ... 173
Spring Diseases ... 228
Samuel Bowles and Dr. Hol[illegible]n[illegible] ... 448

### T

PAGE.

The Gulf Stream............................ 20
The Mouth as a Pocket.................... 21
The Drunkard (poetry)..................... 22
Tom Hunt. A story.......................... 23
The Foster Brothers........................ 52
The Hard Road to Greatness............. 64
The Feet.......................................... 74
Tomatoes.........................................101
The Genus Homo.............................104
The Power of Humbug......................109
The One Leaf (poetry)......................155
To Cure a Cold.................................213
The Earliest Sign of Consumption.....222
The Earth's Great Age......................229
The Catacombs of Paris....................230
The Secret of Good Manners.............2[illegible]5
Taking Comfort in Life......................245
The People Angry.............................246
Truffles............................................248
The Dwarf Diplomat.........................249
The Winning of Wealth.....................250
Treatment of Women........................250
The Nursery Elf (poetry)...................252
The Saragossa Sea............................274
The Vagabond Sage...........................296
The Teeth..........................................307
The Good Old Times.........................327
The Milky Way..................................331
The Emotions....................................352
The Mystery of Color Photography....360
The Best Part of a Man's Life............367
Tea and Coffee..................................380
The Nature of Cough.........................384
The First Sign of Consumption..........387
The Moon and the Weather...............393
The Case of President Garfield.........405
The End of a Fortune........................412
The Utilities......................................413
Throat Oil.........................................415
The Manners of Nations....................429
To Sweep and Dust...........................431
To Our Readers.................................441
Taking Cold......................................442
Tinkering the Postal Laws.................474
True Teachings.................................447
To Prevent Hair Turning Gray...........450
The Toys (poetry).............................447

### U

Uncle Esek's Wisdom.......................155

### V

Ventilation.......................................39
Ventilation.......................................389

### W

Whooping Cough................................6
Western Texas as a Health Resort.......31
Waste of Horse Life..........................71
Water as a Drink...............................165
Wealth and Meanness........................166
Washington's State Dinners...............169
Wakefulness......................................187
What Is? (poetry)..............................191
Watering Horses................................196
When Began We?..............................218
Who Need Eye Glasses?....................230
Women With Moustaches..................275
Why Persons Snore...........................353
When to Feed Grain to Horses...........358
Water From the Rock........................359
Whence They Came..........................432
Words of Courage.............................434

### Y

Yankies in Russia.............................269
Youth and Age (poetry)......................270

